# SEOM-AEEMT consensus on occupational cancer and cancer-associated disability

**DOI:** 10.1007/s12094-025-04037-2

**Published:** 2025-09-16

**Authors:** Laura Mezquita, Mª Teófila Vicente-Herrero, Patricia Cruz, Mª Victoria Ramírez Íñiguez de la Torre, Julia Hidalgo-Coloma, Luisa Capdevila García, Oscar Gallego, Aitana Calvo, Katerine Martínez, Javier Pérez-Altozano, Raquel Molina, Miguel García-Pardo, Laura Gutiérrez-Sainz, Elena Moreno-Atahonero, Martín Oré-Arce, César Serrano, María Jesús Terradillos-García, María Rosario Valero, Luís Reinoso-Barbero, César A. Rodríguez, Beatriz Calvo-Cerrada

**Affiliations:** 1 Medical Oncology Department, Hospital Clinic of Barcelona, Barcelona, Spain; 2 Environmental and Occupational Cancer Working Group, Spanish Society of Medical Oncology (SEOM), Madrid, Spain; 3 Laboratory of Translational Genomics and Targeted Therapies in Solid Tumors, August Pi i Sunyer Biomedical Research Institute (IDIBAPS), Barcelona, Spain; 4 Department of Medicine, University of Barcelona, Barcelona, Spain; 5 Functional Group on Cancer and Work-Asociación Española de Especialistas en Medicina del Trabajo, Grupo de Investigación ADEMA SALUD, Instituto Universitario de Investigación en Ciencias de la Salud (IUNICS), Palma, Islas Baleares Spain; 6 Medical Oncology Department, Hospital Universitario de Ciudad Real, Ciudad Real, Spain; 7 Functional Group on Cancer and Work-Asociación Española de Especialistas en Medicina del Trabajo, Servicio de Prevención, Grupo Correos Albacete, Albacete, Spain; 8 Medical Oncology Department, Hospital Lluís Alcanyís, Xàtiva, Spain; 9 Functional Group on Cancer and Work-Asociación Española de Especialistas en Medicina del Trabajo, Servicio de Prevención Mancomunado MAPFRE, Valencia-MAPFRE Valencia, Valencia, Spain; 10 Medical Oncology Department, Hospital Sant Pau, Barcelona, Spain; 11 Medical Oncology Department, Hospital U. Gregorio Marañón, Madrid, Spain; 12 Medical Oncology Department, IMED Hospitales, Elche, Spain; 13 Medical Oncology Department, Hospital U. Príncipe de Asturias, Alcalá de Henares, Madrid, Spain; 14 Medical Oncology Department, Hospital Ramón y Cajal, Madrid, Spain; 15 Hospital Universitario La Paz, IdiPAZ, Madrid, Spain; 16 Department of Health Promotion and Occupational Epidemiology, Instituto Nacional de Seguridad y Salud en el Trabajo, Madrid, Spain; 17 Medical Oncology Department, Hospital Marina Baixa, Villajoyosa, Spain; 18 Medical Oncology Department, VHIO, Barcelona, Spain; 19 Subdirección General de Coordinación de Unidades Médicas, Instituto Nacional de Seguridad Social, Madrid, Spain; 20 Departamento de Ciencias de la Salud, Universidad Internacional de la Rioja (UNIR), Asociación Española de Especialistas en Medicina del Trabajo (AEEMT), Madrid, Spain; 21 Spanish Society of Medical Oncology (SEOM), Medical Oncology Department, Hospital Universitario de Salamanca-IBSAL, Salamanca, Spain; 22 Functional Group on Cancer and Work-Asociación Española de Especialistas en Medicina del Trabajo, Servicio de Prevención y Salud Laboral Mancomunado Instituto Catalán de Oncología-IDIBELL, Barcelona, Spain

**Keywords:** Occupational cancer, Occupational medicine, Occupational risk factor, Oncology, Occupational carcinogen, Disability

## Abstract

Cancer is one of the main causes of morbidity and mortality in Spain and has a significant impact in the workplace, where exposure to carcinogens in the work environment can increase the risk of developing this disease. The lack of communication between oncologists and occupational physicians limits the accurate assessment of cancer as an occupational disease and as a cause of disability. In 2020, the Spanish Society of Medical Oncology (SEOM) and the Spanish Association of Occupational Medicine Specialists (AEEMT) launched a joint initiative to strengthen prevention, reporting, and management of occupational cancer. This consensus provides a structured framework for assessing the occupational origin of cancer, facilitating case notification and classification as occupational contingencies, and supporting informed evaluations of disability and work reintegration in patients with cancer. The collaboration between both societies aims not only to advance occupational cancer prevention but also to promote evidence-based strategies for return-to-work planning.

**Supplementary Information:**

The online version contains supplementary material available at 10.1007/s12094-025-04037-2.

## Introduction

Cancer is a major public health issue and one of the leading causes of morbidity and mortality worldwide and in Spain. In 2025, approximately 296,103 new cases will be diagnosed, with projections reaching 341,000 by 2040 [1].

Beyond its health impact, cancer significantly affects various aspects of patients’ lives, particularly their work environment. Occupational exposure to carcinogenic risk factors may increase the likelihood of developing work-related cancers. The International Agency for Research on Cancer (IARC) [2] assesses and classifies substances and agents based on their carcinogenic potential in humans. In the workplace, exposure may involve chemical, physical, or biological carcinogens, and preventive action can help reduce or eliminate such risks [3].

Functional limitations due to the disease itself or to long-term adverse effects of anticancer treatments may restrict work capacity, result in disability, or lead to permanent or temporary incapacity.

Limited knowledge and weak communication between occupational physicians and oncologists hinder the integration of cancer-specific information into the assessment of work ability, especially during return-to-work processes. Clear coordination protocols and interdisciplinary collaboration would improve prevention, monitoring, return-to-work support, and notification of cancer as an occupational disease (OD).

Understanding occupational risk factors is essential not only for cancer prevention but also for determining potential entitlements to patient benefits. Oncologists and occupational physicians can play a key role in guiding both healthcare and labor-related processes following a cancer diagnosis. Establishing clear protocols and promoting interdisciplinary collaboration are critical to ensuring comprehensive and appropriate care throughout all phases of the disease and recovery. Additionally, implementing protective strategies for workers and patients is vital to improve current outcomes and prevent occupational cancer.

In 2020, the Spanish Society of Medical Oncology (SEOM) created the *“SEOM Occupational and Environmental Working Group”* to raise awareness, generate knowledge, and promote research in this field. In that same year, the Spanish Association of Occupational Medicine Specialists (AEEMT) established the *“Functional Group on Cancer and Work”* to enhance occupational cancer prevention, generate and share knowledge, and collaborate with other scientific societies to support reporting of occupational cancers.

SEOM and AEEMT have worked together since 2020 under a formal agreement aimed at generating knowledge and fostering an integrated approach to occupational cancer. This SEOM-AEEMT consensus emphasizes the role of cancer as an OD and a leading cause of both temporary and permanent work disability in Spain. It outlines criteria for determining occupational causality, procedures for case reporting and classification as occupational contingency, and coordinated action algorithms, along with future strategies to strengthen occupational cancer prevention.

## Occupational cancer

### Context and recognition of occupational cancer

Cancer is the leading cause of work-related mortality in the European Union and is often linked to occupational exposure to carcinogens [4]. Around 120,000 cancer cases in Europe each year are attributed to workplace exposure, with nearly 80,000 resulting deaths. In Spain, estimates suggest 6,500–13,500 new cases annually are related to occupational risks [5], yet only 0.10–0.22% are formally recognized as occupational diseases (OD), significantly below the 4% estimated by Doll and Peto [6].

The Spanish General Social Security Law (LGSS) identifies two categories where work activity can impact health: occupational accidents (OA) and occupational diseases [7]. To classify cancer as an OD, it must appear in Annex I of Royal Decree 1299/2006, which lists—not exhaustively—recognized diseases, their causal agents, and related occupational activities [8]. Cases officially recognized as OD are reported through the CEPROSS system [9]. Cancers not listed but with demonstrated work-related causality are classified as "work-related diseases" and reported through PANOTRATSS [9].

Underreporting of occupational cancer is influenced by limited awareness of occupational risks, insufficient training among oncologists to notify ODs, and bureaucratic complexity. The lack of coordination between occupational medicine and oncology further complicates this issue by limiting integrated clinical and occupational decision-making in the management and post-treatment reintegration of affected patients. Consequently, workers with occupational cancer may be excluded from national surveillance systems and denied access to the social protections and benefits established for OD under the Spanish Social Security framework.

### Exposure to carcinogens in the workplace

A carcinogen is defined as any agent with sufficient evidence of a causal link between human exposure and cancer development [10]. The WHO identifies 47 occupational carcinogens and 12 industries occupations linked to an increased cancer risk among workers [11]. In Spain, Royal Decree (RD) 612/2024 [12], which updates RD 427/2021 [13], regulates the protection of workers against risks derived from exposure to carcinogenic, mutagenic, or reprotoxic agents in the workplace and establishes the minimum specific provisions that must be applied in activities involving exposure to such agents. Supplementary Table 1 summarizes occupational exposure limit values according to RD 612/2024.

To recognize cancer as an OD in Spain, three criteria from the LGSS must be met: (a) the disease must be contracted as a result of the work performed, (b) the job responsible for the exposure must be listed in the occupational disease schedule, and (c) the disease must be caused by the elements or substances indicated for each OD in that schedule. Accordingly, malignancies caused by carcinogens listed in Group 6 of Annex 1 of RD 1299/2006 are recognized as ODs and criteria for notification and registration are established [8]. This RD has been updated through RD 1150/2015 [14] and RD 257/2018 [15]. Table 1 presents the list of carcinogenic agents, the tumors associated with occupational exposure, and the industrial activities in which these ODs are recognized.

**Table 1:** Occupational diseases (OD) related to exposure to carcinogenic agents and occupational activities according to Royal Decrees 1299/2006, 1150/2015, and 257/2018

Occupational cancers according to Spanish Royal Decree	Carcinogen agent	Occupational activity (not exhaustive list)
RD 1299/2006		
Malignant neoplasm of bronchus and lung	• Asbestos • Arsenic and its compounds • Beryllium • Bis(chloromethyl) ether • Cadmium • Chromium VI and its compounds • Nickel and its compounds • Radon • Free silica dust	Extraction, handling, and processing of asbestos-containing minerals or rocks; manufacturing of asbestos fabrics, cardboard, and paper; preparatory treatment of asbestos fibers; car repair works; dismantling and demolition of facilities containing asbestos. Handling and use of arsenic and its compounds. Work involving beryllium and its compounds (e.g., beryllium fluoride), particularly in beryllium extraction and metallurgy, aerospace and nuclear industries, crystal and ceramics production. Production of plastics and vulcanized rubber. Industrial preparation and use of cadmium. Use of hexavalent chromium compounds in catalyst and pigment production, tanning, and wood treatment. Nickel smelting and refining, stainless steel manufacturing, battery production. Work involving pigments, chimney soot removal, road paving, insulation, cable production, aluminum processing, and driving occupations. Underground mining and processes involving natural uranium decay products (Radon-222). Mining, tunneling, quarrying, and ceramic-related industries involving silica exposure
Mesothelioma	• Asbestos	Extraction, handling, and processing of asbestos-containing minerals or rocks; manufacturing of asbestos fabrics, cardboard, and paper; preparatory treatment of asbestos fibers; car repair works; dismantling and demolition of facilities containing asbestos
Malignant neoplasm of the bladder	• Aromatic amines • Primary, secondary, tertiary, heterocyclic amines • Aromatic hydrazines and their halogenated, phenolic, nitrosated, nitrated, and sulfonated derivatives	Manufacture and use of aromatic amines; rubber industry workers; use of dyes containing β-naphthylamine, benzidine, amino-diphenyl, nitro-diphenyl, auramine, magenta, and their salts; and their intermediates in synthetic dye, chemical, insecticide, and pharmaceutical industries
Malignant neoplasm of the prostate	• Cadmium	Industrial preparation and use of cadmium
Squamous cell carcinoma of the skin	• Arsenic and its compounds • Ionizing radiation	Handling and use of arsenic compounds; exposure to X-rays or radioactive substances from any natural or artificial source
Squamous cell carcinoma	• Polycyclic aromatic hydrocarbons (PAHs) • Coal distillation products (soot, tar, pitch, anthracene, mineral oils, raw paraffin), and related residues	Pigment manufacturing, chimney cleaning, road paving, insulation, electrical cable production, asphalt handling, vehicle maintenance, and work in combustion units
Bowen's disease (discoid lenticular dyskeratosis)	• Arsenic and its compounds	Handling and use of arsenic compounds
Lymphoproliferative and myeloproliferative syndromes	• Benzene • Ionizing radiation	Benzene manufacture, use and purification; exposure to ionizing radiation from any natural or artificial source
Lymphoma	• Nitrobenzene	Solvent use; production of dyes and pigments
Malignant neoplasm of the liver and intrahepatic bile ducts	• Vinyl chloride monomer	Production and polymerization of vinyl chloride
Hepatic angiosarcoma	• Arsenic and its compounds • Vinyl chloride monomer	As previously described for arsenic and vinyl chloride
Malignant neoplasm of the nasal cavity	• Chromium VI compounds • Nickel compounds • Hardwood dust	Handling and use of hexavalent chromium compounds in catalyst, dye, pigment production, tanning, and wood treatment; nickel smelting and refining, stainless steel manufacturing, battery production; work involving hardwood
Primary cancer of the ethmoid and paranasal sinuses	• Nickel and its compounds	Nickel refining, stainless steel production, battery manufacturing
RD 1150/2015		
Malignant neoplasm of the larynx	• Asbestos	Industries using asbestos; manufacture of brake pads, clutches, fiber cement products; vehicle repairs; dismantling and demolition of asbestos-containing structures
RD 257/2018		
Malignant neoplasm of the bronchus and lung	• Free silica dust	Mining, tunneling, quarrying, public works, and the production of carborundum, glass, porcelain, ceramics, and silica-based refractory bricks

To classify a cancer as either an occupational disease or work-related accident, several criteria must be fulfilled: (a) the diagnosis must align with the health effects associated with exposure to a specific agent, (b) sufficient occupational exposure must be demonstrated before disease onset, (c) the latency period must match what is known about the natural history of the disease, and (d) differential diagnosis must exclude similar diseases caused by non-occupational exposures [16]. Key elements in establishing a plausible link include exposure intensity and duration, latency, and identification of specific workplace agents. Health professionals must assess these carefully to ensure accurate notification. Distinctive features of occupational tumors include: (a) Higher incidence in younger individuals (especially when exposure occurred early in the professional career); (b) Clustering in groups of workers with similar occupational exposure; and (c) Greater likelihood of occurrence in the context of co-exposure to other carcinogens, occupational or not [11].

Determining the occupational origin of cancer in a worker may be complex due to several reasons:

- Both occupational and non-occupational risk factors may coexist. Co-exposure to external factors such as tobacco or alcohol does not exclude the causality of an occupational carcinogen in the development of the disease.
- Occupational cancers may present identical histopathological features to those of non-occupational origin.
- It is often difficult to determine whether workplace exposure triggered the carcinogenic process or contributed to tumor progression.

All potential sources of exposure must be evaluated to ensure proper recognition and treatment of occupational cancer. In Spain, there are initiatives such as the CEPS project [17], aimed at evaluating the burden of occupational diseases, including cancer, treated in hospitals.

Declaring an OD provides several legal and financial protections:

1. No prior contribution period is required to qualify for disability benefits or pension calculations.
2. Full coverage for medical assistance, including medications, rehabilitation, reconstructive surgery, prosthetics, and transportation, all funded by the managing entity (INSS, mutual collaborator, or self-insured employer).
3. In the event of death, the closest family members are entitled to financial compensation.
4. Advantages in economic benefits for the affected individual: (a) temporary disability (TD): a subsidy equivalent to 75% of the regulatory base from the second day of TD (the first day is covered by the employer) until the end of the TD period; (b) permanent disability (PD): a lifelong pension in cases of irreversible incapacitating injury; (c) possibility of increasing all benefits by 30–50% if the company failed to implement proper preventive measures. (d) Additional compensation in certain cases.
5. Death and survivor protection, including benefits for dependents of the deceased worker.

### Procedure for declaring cancer as an occupational disease: action algorithm

The article 5 of RD 1299/2006 [8] states that all healthcare professionals within the Spanish National Health System (SNS), as well as occupational health services of companies or organizations, are required to report suspected OD for official recognition, via the competent authority in each Autonomous Community (CCAA).

Currently, medical oncologists rarely report such suspicions, largely due to the factors previously described. Most notifications of suspected occupational cancer cases from the healthcare setting originate in primary care.

However, medical oncologists, like other hospital-based physicians, have a formal responsibility to report suspected occupational cancer. Given their exclusive focus on patients with cancer, including those potentially linked to occupational exposures, it is essential that they are familiar with reporting procedures and contribute to the recognition of cancer cases with a potential occupational origin.

**Fig. 1:**
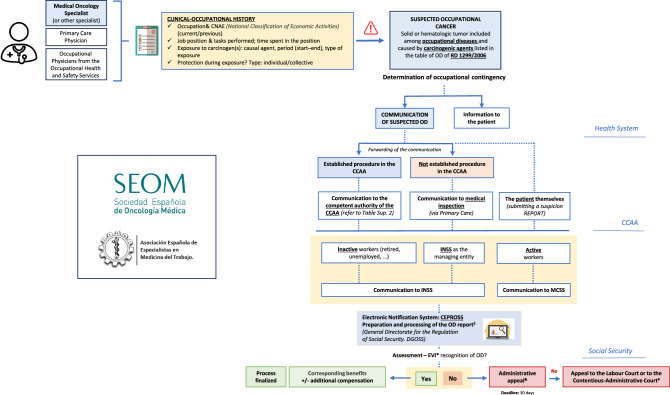
Algorithm for notification of suspected and declared occupational cancer. According to Royal Decree 1299/2006^2^. As provided in RD 1299/2006, RD 1150/2015, RD 257/2018. CCAA (Autonomous Communities), INSS (National Institute of Social Security), MCSS (Mutual Insurance Collaborating with Social Security), CEPROSS (Communication of Occupational Diseases, Social Security), DGOSS (General Directorate of Social Security Planning). EVI (Disability Assessment Team). *The EVI only intervenes in cases where there is a procedure for determining the contingency that is not recognized by the managing entity (INSS or Mutual Insurance). ^#^Prior claim to the INSS. ^+^To the Mutual Insurance Company or the INSS, depending on: - If the worker is active: to the entity with which the company has contracted professional contingencies (Mutual Insurance, INSS, ISM). - If the worker is retired or unemployed: to the INSS, by requesting a "Determination of Contingency". ^&^Only if the Mutual Insurance Company does not recognize the occupational nature of the disease. Before appealing in court, an administrative claim must be submitted to the competent authority (INSS or the Mutual Insurance Company) within 30 days from the date the denial resolution is received

When a medical oncologist, another SNS hospital-based physicians, or an occupational health professional becomes aware of a disease listed as an occupational disease or one that is suspected to be occupational in origin, they must report it through the relevant CCAA authority [18]. Figure 1 outlines the steps involved in reporting suspected occupational cancer and the corresponding declaration algorithm.

Some CCAAs have specific procedures in place for reporting suspected occupational diseases. In other regions, the report can be submitted to the institution responsible for managing occupational contingencies (e.g., Mutual Collaborating Entities with Social Security [MCSS], National Institute of Social Security [INSS], or collaborating employer) via the intermediary authority designated by each CCAA:

1. The hospital-based physician submits a suspicion report directly to the competent CCAA authority, which instructs the MCSS, INSS, or collaborating employer to initiate an investigation for possible OD recognition.
2. The suspicion may also be reported via the primary care physician, who prepares a report for the local medical inspector and provides a copy to the patient. The patient submits this report to the MCSS to initiate the diagnostic and treatment process, while the inspector can inform the INSS.
3. The occupational physician from the SP or Occupational Health Unit of the respective CCAA is required to notify the competent entity within three business days of diagnosing a suspected OD.

This reporting must follow the guidelines of Order TAS/1/2007 [19], which defines the official reporting form, the standards for its preparation and transmission, and the corresponding personal data file. However, not all CCAA have implemented a dedicated notification system or specific legislation to standardize the reporting of suspected occupational diseases. Table 2 summarizes the availability and characteristics of regional notification procedures across different CCAAs.

**Table 2:** Process for reporting suspected occupational disease by Autonomous Community (AC)

Autonomous Community	Notification system and regional responsibility	Competent authority by AC	How to perform the procedure?
Andalusia	Not established, no specific legislation in the Autonomous Community	Consejería de Salud	Not established; refer the patient through Primary Care and the medical inspection
Aragón	Not established, no specific legislation in the Autonomous Community	Consejería de Salud	Not established; refer the patient through Primary Care and the medical inspection
Islas Baleares	Not established, no specific legislation in the Autonomous Community; Instituto Balear de Seguridad y Salud Laboral (IBASSAL)	Consejería de Salud	Primary Care.or mutual insurance provider Option available via online platform (https://www.caib.es/seucaib/es/tramites/tramite/3771638)
Canarias	Not established, no specific legislation in the Autonomous Community; Unidad de Salud Laboral y Atención Primaria	Consejería de Salud	Detailed information available on the web contact point (see Table 2—supplementary material)
Cantabria	Instituto Cántabro de Salud y Seguridad en el Trabajo	Instituto Cántabro de Seguridad y Salud en el Trabajo (CASST)	Not established; refer the patient through Primary Care and the medical inspection
Castilla-La Mancha	Not established, no specific legislation in the Autonomous Community	Dirección General de Salud Pública. Servicio de Sanidad Ambiental, Salud Laboral y Lab. Del Salud Pública	Not established; refer the patient through Primary Care and the medical inspection
Castilla y León	Not established, no specific legislation in the Autonomous Community	Consejería de Salud	Not established; refer the patient through Primary Care and the medical inspection
Cataluña	Not established, no specific legislation in the Autonomous Community	Consejería de Salud	Not established; refer the patient through Primary Care and the medical inspection
Comunidad de Madrid	Sistema de Notificación de enfermedad profesional	Dirección General de Salud Pública	Detailed information available on the web contact point (see Table 2—supplementary material)
Comunidad Foral de Navarra	Sucesos centinela en Salud Laboral; Programa de IT común; Sospecha de enfermedad profesional	Instituto de Salud Pública y Laboral de Navarra	Not established; refer the patient through Primary Care and the medical inspection
Comunidad Valenciana	SISVEL (Sistema de Información Sanitaria y Vigilancia Epidemiológica Laboral)	Dirección General de Salud Pública. Conselleria de Sanidad Universal y Salud Pública	
Extremadura	Not established, no specific legislation in the Autonomous Community	Dirección General de Planificación, Formación y Calidad Sanitarias y Sociosanitarias. Servicio de Inspección Sanitaria	Not established; refer the patient through Primary Care and the medical inspection
Galicia	Not established, no specific legislation in the Autonomous Community	Dirección Xeral de Saude Pública; Subdirección Xeral de Inspección Auditoria e Acreditación de Sevizos Sanitarios	Not established; refer the patient through Primary Care and the medical inspection
País Vasco	Unidad de Salud Laboral (USL-Osalan-Instituto Vasco de Seguridad y Salud Laborales) e Inspección Médica de Servisios Sanitarios (Consejería de Salud)	Instituto Vasco de Seguridad y Salud Laborales. Departamento de Salud	Medical professionals from Osakidetza and Occupational Health Services
Principado de Asturias	Programa de Detección de Cáncer Laboral (EVASCAP), Servicio de Salud del Principado de Asturias	Dirección General de Salud Pública-EPILABAM	Through Primary Care **Route 1** (less frequent) involves direct suspicion by the healthcare professional, without the patient necessarily being in active employment, via Primary Care (OMI-AP). **Route 2** (more commonly used): Based on sick leave reports, potential cases are identified by the Inspection Service through their regional inspections using an alert system integrated into the IT (temporary disability) management software. The alert system detects IT processes whose diagnoses, coded using the International Classification of Diseases (ICD), match cancer diagnoses listed in the official table of occupational diseases. In such cases, the regional inspection team sends a letter to the physician who prescribed the sick leave, requesting the completion of the patient’s occupational history using a specific form designed for this purpose, which is enclosed with the letter
Región de Murcia	Unidad de Sospecha de Enfermedades Profesionales (USSEP); no legislación específica en la CA	Consejería de Salud	Not established; refer the patient through Primary Care and the medical inspection
La Rioja	Not established, no specific legislation in the Autonomous Community; Salud Laboral	Salud Laboral, Dirección General de Salud Pública y Consumo	Not established; refer the patient through Primary Care and the medical inspection

The official occupational disease form is submitted electronically through the CEPROSS system [9]. As mentioned before, for cancers not listed but with demonstrated occupational causality are classified as "work-related diseases" and reported through the PANOTRATSS system [9]. The competent institution for determining the contingency status (i.e., whether the cancer is recognized as an OD) is the INSS. Upon collecting the necessary documentation, the INSS issues a formal resolution. If the disease is not recognized as occupational, the patient may appeal the decision via administrative or judicial proceedings.

### SEOM-AEEMT group perspective

- Spanish legislation establishes that all physicians within the SNS have the responsibility to report suspected occupational cancer to the competent authority of each CCAA. Education, awareness, and involvement of medical oncologists in this reporting process are essential to improve current underreporting of occupational cancers in Spain.
- Underreporting of OD leads to underestimation and inadequate registry of cases with suspected work-related etiology. A lack of knowledge among hospital and primary care physicians, along with regional variability in reporting procedures, contributes to many cases not being properly identified or declared as OD.
- Accurate reporting of occupational cancer requires the active involvement of all professionals—both clinical and occupational—who can initiate the suspicion and collaborate in the subsequent investigation needed to classify it as an OD.
- A coordinated effort is needed between healthcare professionals, medical societies, and occupational health authorities. Close collaboration between clinical physicians—specialized in diagnosis, treatment, and monitoring of cancer—and occupational medicine physicians—with a preventive role—is essential.
- Awareness and training strategies targeting physicians across specialties are needed, with a particular emphasis on the involvement of medical oncologists in the notification process. Understanding administrative procedures related to occupational disease declaration is key. Only a multidisciplinary, coordinated approach can improve identification and recognition of work-related cancer.
- Knowledge of OD affecting workers allows for more effective prevention strategies and early diagnosis, enabling timely treatment, particularly relevant in cancers where earlier detection is associated with higher cure rates.
- Recognition of the occupational origin of cancer has legal, administrative, and financial implications, and it ensures patients’ access to the benefits and protections established for OD.

## Cancer as a cause of work disability

### Context and key points

Cancer involves long periods of temporary disability (TD), with an average duration of 280 to 365 days. In 90% of cases, TD lasts over 6 months. Although survival has improved—about 53% of patients survive more than five years—neoplasms account for 10% of permanent disability (PD) declarations. Over 50% of cancer patients are granted some form of PD. Additionally, 30% of survivors lose their job and 55% do not return to work [20].

Medical oncologists play a key role in managing work disability in patients with cancer. They provide medical information on tumor characteristics, stage, chronic side effects, functional limitations, sequelae, and prognosis. Often, they provide medical reports detailing treatment history and justifying work leave and can offer additional data into residual functional limitations. This information supports occupational medicine physicians in assessing work fitness, identifying limitations, and determining whether job accommodations are needed to ensure a safe return to work.

### Definition and types of work disability

Work disability concepts are defined by the LGSS, which also regulates the eligibility criteria for financial benefits [7]. These processes have a significant economic impact, particularly for the most prevalent cancers, regardless of whether they are occupational in origin, and may be worsened by job-related strain. Table 3 summarizes the types of disability, corresponding levels, and benefits.

**Table 3:** Summary of types of disability, degree of disability, and type of benefit defined by the LGSS

Type of disability	Degree	Key information	Type of benefit
Temporary disability (TD)	Not applicable	• Illness or accident (common or occupational) that prevents work activity • Maximum duration: 365 days + 180-day extension • Presumed potential for recovery/improvement enabling return to work	Contributory, if applicable
Permanent contributory disability (PD)	Partial	• Irreversible reduction in work capacity of at least 33% • Legally compatible with regular duties and other activities • Subject to review upon health status change	Contributory, if applicable
	Total	• Irreversible impairment preventing fundamental tasks of regular profession • Lifelong character • Possibility to engage in different work activity	Contributory, if applicable
	Absolute	• Irreversible inability to perform any work activity • Incompatible with any paid employment	Contributory, if applicable (maximum amount: 100% of the regulatory base if affiliation conditions are met)
	Severe disability	• Maximum degree of irreversible physical and functional incapacity • Requires permanent assistance • Prevents any work activity, needs help with daily activities	Contributory, if applicable (maximum amount: 100% of regulatory base with supplement for basic assistance)

Among the recognized forms of work disability in the Spanish legal framework are the following:

- *Temporary disability (TD)* This status applies to workers temporarily unable to perform their job due to OD or common disease or accident (work-related or not) while receiving treatment from the Social Security system. TD can last up to 365 days, extendable by 180 days if recovery is expected. It also includes observation periods for suspected OD, initially for 6 months and extendable another 6 months for diagnostic evaluation. This type of disability is potentially reversible, allowing for recovery of work capacity within a maximum period of 545 days [21]. Regulations governing TD, including its management and associated benefits, are specified in Article 169 of the LGSS [14]. Supplementary Fig. 1 summarizes the roles and responsibilities related to temporary disability processes (LGSS). TD duration can be influenced by biological, social, cultural, and healthcare-related factors. In cancer, assessing these factors is complex. INSS manuals provide estimated TD durations [22], based on consensus from various medical societies across specialties [23].
- *Permanent disability (PD)* According to RD 8/2015, PD refers to the inability to perform all or essential duties of one’s usual profession due to serious and likely permanent anatomical or functional impairments [7]**.** It may result from worsening preexisting conditions or new complications that reduce work capacity. PD typically follows prolonged TD. It is classified by severity according to irrecoverable loss of work capacity and directly affects contributory benefits. The different types of PD (partial, total, absolute, or severe disability) and related benefits are specified in Article 193 of Chapter XI of the LGSS. Supplementary Fig. 2 outlines the basic PD concepts and levels.
- Other concepts include *non-disabling permanent injuries*, which entitle the worker to a lump-sum compensation for work-related sequelae that do not impair job performance. These are regulated in Articles 201–203 of the LGSS.

### Steps for determining permanent work disability

Based on the LGSS, decisions about disability status are made by the provincial director of the National Institute of Social Security (INSS), based on a proposal from the Disability Assessment Team (EVI), which includes a summary medical report prepared by an INSS inspector. In all Spanish CCAA except Catalonia, this procedure is centralized within the INSS. In Catalonia, however, the process is handled by the Catalan Institute for Disability Evaluation and the Disability Assessment Commission. The PD assessment process can be initiated by the patient or their legal representative, the public health service (via the medical inspector), the MCSS, the Labor Inspectorate, or the INSS.

In cancer, PD assessment is a complex process considering:

1. Characteristics of the affected worker
2. Cancer type and stage at diagnosis
3. Evaluation of contingency: whether there is an occupational exposure relationship
4. Type of treatment and short- and long-term side effects
5. Post-treatment care, follow-up, adaptation, and rehabilitation
6. Occupation and job role. Evaluation of the job position and assessment of potential modifications

INSS medical inspectors undergo specific, ongoing training in disability evaluation and use guidelines [23] to support clinical decision-making. They also consider reports and test results from public health service specialists. Additional evaluations or tests may be requested if necessary. PD certification requires definitive functional limitations and exhaustion of treatment and job adaptation options. In some cases, PD status is granted with a scheduled review period, depending on possible improvement or deterioration due to long and unpredictable disease courses. Chronic treatment in patients with cancer associated with good outcomes does not by itself justify PD; a case-by-case evaluation is needed, weighing the patient’s functional limitations (from disease or treatment) against job requirements.

### Return to work

At diagnosis, most cancer cases are classified as common diseases. Around 98% of employers contract common disease coverage with a MCSS. TD is initially certified by a primary care physician, but follow-up is conducted jointly by the primary care physician and the MCSS, which provides financial support.

TD ends in the following cases:

- *Medical discharge due to improvement or recovery* By the certifying primary care physician, the public health service inspector, or the INSS inspector. Patients may also request discharge if they feel ready to return to work. Within the first 365 days of TD, the PCP handles this; between days 365 and 545, the INSS is responsible.
- *Reaching the maximum 545-day TD limit* Discharge may involve initiating a PD assessment or planning a return to work.
- *Patient death.*

Upon discharge without a PD declaration, the worker must return to work. Following prolonged TD, the worker is evaluated by the occupational medicine physician of the company's prevention service to identify potential occupational causes of disease, assess compatibility between clinical status and job requirements, and, if necessary, recommend appropriate adaptations, as outlined in Article 37 of the Spanish Occupational Risk Prevention Regulations [24].

After return to work, an "overcome unfitness" situation may occur: a condition in which a worker, after TD, is no longer fit to perform their contracted job duties, with the impairment arising after employment began [25]. This may lead to contract termination for objective reasons, in accordance with Article 52 of RD 2/2015 [14].

Special consideration is given to "especially sensitive" workers, per Article 25 of Law 31/1995 on Occupational Risk Prevention; employers must provide specific protections, based on a report from an occupational medicine physician [26].

Information provided by oncologists in reports for the INSS or workplace prevention services is fundamental. These reports should include the cancer diagnosis, initial functional status, prognosis, treatment details and side effects, and any long-term sequelae.

Details about treatment, toxicity, and functional status after therapy are key for occupational medicine physicians to assess job fitness, based on whether the worker can safely carry out job tasks without endangering themselves or others. Occupational medicine physicians should also recommend necessary job adaptations and preventive measures to be implemented by company prevention services.

Table 4 presents a model of structured information that oncologists should include in medical reports for occupational health professionals and medical inspectors to assist in TD and PD decision-making processes.

**Table 4:** Structured Medical Report model with key information the oncologist may include in a medical report for Occupational Health and/or Medical Inspectors/INSS to support the assessment of Work Fitness and/or the type and degree of disability in patients with cancer

Medical report template to be completed by the Medical Oncologist for Occupational Medicine and/or Medical Inspectors/INSS	
Section	Relevant information
Patient information	Age Gender and sex Relevant past medical diagnoses
Cancer diagnosis	Tumor type and location Date of diagnosis Stage at diagnosis
Treatment details	Detailed description of treatments received (surgery, radiotherapy, chemotherapy, immunotherapy, etc.) Start and end dates of each treatment Treatment response
Treatment side effects	Acute side effects Chronic (long-term) side effects Impact of side effects on the patient’s quality of life
Functional status	Performance Status (PS), using ECOG and/or Karnofsky scale Specific functional limitations (mobility, fatigue, pain, etc.) Ability to perform daily living activities
Sequelae related to the disease and/or treatment	Description of permanent sequelae Severity of sequelae
Prognosis	Risk of recurrence Estimated survival
Rehabilitation and adaptive recommendations	Need for physical and/or psychological rehabilitation Job-related limitations (if occupational background is known)
Other relevant factors	Comorbidities that may impact work capacity Emotional and psychological status of the patient
Additional information	Reports from other specialists (if applicable) Results from diagnostic tests

### SEOM-AEEMT group perspective

- Cancer contributes significantly to work disability. In 90% of cases, TD exceeds six months, and over half progress to PD. Therefore, proactive collaboration between oncologists and occupational medicine physicians is key. Oncologists should offer clinical support and prepare detailed reports to support occupational assessments and job adaptations. Shared clinical and work histories are highly relevant to facilitate safe and sustainable return to work.
- TD and PD follow specific LGSS criteria:
- TD can last up to 545 days if recovery is expected. This extended duration carries significant social and healthcare costs.
- PD is classified into several degrees based on the irreparable loss of work capacity, determining the financial benefits established by regulations.
- Returning to work after cancer-related TD is complex. It requires work fitness assessments and, often, job accommodations aligned with the Spanish legislation. Close medical monitoring is critical to ensure a safe and sustained return to work.
- Rising cancer survival, due to early detection, prompt treatment, and improved therapies, supports return to work. This process must consider potential post-treatment sequelae and toxicities, guiding necessary limitations or adaptations.

## Future perspectives and recommendations

Based on the previous sections, there is a clear need to implement actions to improve the current situation regarding cancer as an OD and as a cause of work disability or impairment. Medical oncologists, occupational medicine physicians, and other professionals share a responsibility and commitment to promote feasible and transformative measures in the short and medium term.

### Multidisciplinary strategy in cancer and work

Reducing the underreporting of occupational cancer in Spain requires close collaboration among medical oncologists, occupational medicine physicians, and primary care medicine specialists through the creation of integrated multidisciplinary teams. This collaboration would enable the identification of occupational cases, promote their recognition as ODs, and improve awareness of occupational risk exposures, with significant implications for prevention.

These teams would provide support for the patients and affected workers through the administrative steps required to initiate OD declarations, offering effective and timely responses in complex cases. This ensures comprehensive support during diagnosis, treatment, recovery, and safe return to work.

SEOM and AEEMT have collaborated since 2020 on a joint project to enhance awareness, knowledge, and research on occupational cancer in Spain. This partnership includes training initiatives, development of clinical protocols, and promotion of preventive strategies to ensure comprehensive and high-quality care for patients with occupational cancers. The SEOM and AEEMT Working Groups are committed to improving knowledge and response capacity regarding occupational cancer in Spain.

Our objective is twofold: first, to promote policies and practices that ensure a safe and healthy work environment for individuals at risk of cancer, particularly those who have not yet developed the disease. Second, for patients with cancer, to enhance the identification and appropriate recognition of OD when applicable, and to support the assessment of disability, follow-up, workplace adaptation, and job reintegration, regardless of the cancer's origin. Both groups also actively contribute to the Spanish Strategy for Safety and Health at Work (2023–2027) convened by the National Institute for Occupational Safety and Health (INSST) [27], which prioritizes occupational cancer through working groups on early diagnosis, intervention, and epidemiology.

### Improved documentation of occupational history

A complete clinical history that includes the patient’s occupational background is mandatory to suspect and diagnose occupational cancer. Implementing a standardized occupational history model that collects essential and relevant data useful to both specialties aims to support early identification of occupational cancer and the initiation of the OD recognition process [28].

Table 5 summarizes the proposed occupational information that should be included in the medical history of patients with cancer, according to the SEOM-AEEMT Working Group. Collecting this data during the initial consultation may help identify potential OD cases.

**Table 5:** Proposed occupational history information for cancer patients that could be collected during the initial oncology evaluation to assess the suspicion of an occupational disease (OD)

Occupational history (to be integrated with the oncology clinical history)	Information to be collected to assess the communication of suspected occupational disease
Current job information	• Profession and economic sector • Information on current job position: - Job according to CNAE - Tasks performed - Workplace - Duration in months, start/end date • Is the job related to a suspected occupational disease (RD 1299/2006)?
Previous employment information	• Information on patient’s previous jobs, including dates
Exposure to carcinogens in current and previous jobs	• Exposure to any known carcinogen at work? - Duration of exposure in months, start/end date - Safety measures and protective equipment: individual/collective? • Description of exposures to potential carcinogens at work
Previous diagnoses	• Information on non-oncological work-related diseases
Employment data	• Company(ies) and workplace(s) • Collaborating Mutual Insurance Company with the Social Security
SUSPECTED diagnosis	• Diagnosis: ICD code (recommended, 4 digits) • Date of diagnosis • Tumor type (histology) and other relevant information

### Integrating medical oncologists into the occupational cancer reporting process

Underreporting of OD in Spain is an urgent challenge requiring an effective approach through collaboration among medical societies. Joint efforts should focus on continuous education, awareness campaigns, and dissemination of practical guidelines to aid public health professionals in reporting suspected cases. Practical support tools and consensus-based guidelines, such as those proposed in this document, can facilitate this process.

Advancing OD reporting requires the transmission of relevant information to initiate the investigation process. A key example of progress is the prospective study “CENTINELA 01: Sentinel Medical Oncologist Network for Identifying Occupational Cancer in the National Health System”, led by the “SEOM Occupational and Environmental Working Group”, in collaboration with the “Head and Neck Tumor Treatment Group” (TTCC) and promoted by the INSST. CENTINELA 01 aligns with Objective 1 of the Spanish Occupational Safety and Health Strategy. Active since March 2024, this pilot study was conducted in 23 centers across 12 CCAAs, prospectively enrolling patients with sinonasal tumors. Recruitment concluded in February 2025, and the results are currently under analysis and will be reported soon.

One of the top priorities of the SEOM Working Group is to raise awareness among medical oncologist about the increasing relevance of occupational cancer. Specific actions include organizing a dedicated sessions at the SEOM 2023 National Congress and launching training sessions in Hospitals from CCAAs with high occupational cancer prevalence. Awareness campaigns, combined with pilot projects like CENTINELA 01, are already contributing significantly to the necessary change.

### Research and development

Advancing the identification and understanding of requires a sustained emphasis on both epidemiological and oncological research. This is a dynamic field, marked by the continuous discovery of new occupational carcinogens and the integration of novel exposures and tumor types into regulatory frameworks. Staying informed and generating new knowledge from clinical practice is key.

Medical oncologists, drawing on patients’ clinical and occupational histories, can serve as clinical investigators, contributing not only to the identification of established occupational cancers, but also to the detection of new associations and the evaluation of emerging or under-recognized risk factors. Collaboration among medical oncologists, occupational medicine physicians, epidemiologists, and other specialists enables the generation of robust evidence, hypothesis development, and the design and execution of meaningful studies.

Within multidisciplinary teams, the integration of oncologists’ expertise in cancer biology allows for the exploration of molecular and immunological pathways and the identification of biomarkers that may link occupational exposures to carcinogenesis. This collective expertise fosters a deeper understanding of disease mechanisms, promotes professional collaboration, and reinforces the scientific impact for both prevention and clinical management.

Moreover, this approach may help support future OD recognition by clarifying the contribution of occupational risk factors in complex, multifactorial cancers. Ultimately, the objective is to establish a global, coordinated, and integrated strategy across healthcare and preventive systems, delivering a 360° perspective on cancer and work [29].

## Conclusion

Occupational cancer remains a significant yet under-recognized issue in Spain. Its dual impact, as a preventable disease associated with workplace exposures and a frequent cause of long-term disability, calls for a coordinated, multidisciplinary approach involving medical oncologist and occupational medicine physicians. This consensus, jointly developed by SEOM and AEEMT, provides a practical framework and shared protocols to bridge medical oncology and occupational health. It emphasized the importance of increasing awareness among oncologists and improving the identification, reporting, and classification of occupational cancer as well as the cancer as cause of disability. These efforts are essential to ensure equitable disability assessments, access to social protection, and support for effective work reintegration.

The strategies outlined here reframe occupational cancer not only as a public health concern, but also as an issue of equity, clinical excellence, and patient rights. Advancing prevention, research, and education in this field will help deliver a more integrated and equitable model of cancer care in Spain.

## Supplementary Information

Below is the link to the electronic supplementary material.

Supplementary file1 (DOCX 281 KB)

Supplementary file2 (XLSX 22 KB)
