# Supplementary material for: SEOM-AEEMT consensus on occupational cancer and cancer-associated disability
Source: Clin Transl Oncol. 2025 Sep 16;28(3):880–94. doi: 10.1007/s12094-025-04037-2 (PMC12920288; doi:10.1007/s12094-025-04037-2)
Supplement: Supplementary file 1 — Supplementary file1 (DOCX 281 KB) [file 12094_2025_4037_MOESM1_ESM.docx]

**SUPPLEMENTARY LIST AND TABLES:**

**Supplementary Table 1.** Occupational exposure limit values. (1) CE Number, i.e., EINECS, ELINCS, or "no-longer polymer" (NLP), is the official number of the substance in the European Union, as defined in section 1.1.1.2 of Annex VI, Part 1, of Regulation (EC) No. 1272/2008. (2) CAS Number: Chemical Abstracts Service registry number. (3) Measured or calculated as a time-weighted average over an eight-hour reference period. (4) Short-term exposure limit: the limit value above which exposure should not occur, referring to a 15-minute period unless otherwise specified. (5) mg/m³ = milligrams per cubic meter of air at 20 °C and 101.3 kPa (760 mm Hg). (6) ppm = parts per million by volume of air (ml/m³). (7) f/ml = fibers per milliliter. (8) Inhalable fraction: if hardwood dust is mixed with dust from other woods, the limit value applies to the total wood dust present in the mixture. (9) Respirable fraction. (10) Potential significant contribution to total body burden via dermal exposure.

**Supplementary Table 2.** Process for Reporting Suspected Occupational Disease by Autonomous Community (AC).

**Supplementary Figure 1. Responsibilities in Temporary Disability Processes.**Source: General Social Security Law (LGSS). *INSS: National Institute of Social Security. Legend: Temporary Disability (TD); National Institute of Social Security (INSS); Public Health Service (SPS).

**Supplementary Figure 2. Basic concepts and degrees of permanent disability.**
Source: Royal Decree 8/2015 of October 30, which approves the revised text of the General Social Security Law (Official State Gazette No. 261, October 31, 2015. Reference: BOE-A-2015-11724).
a Permanent disability generally arises from a prior period of temporary disability, except in very specific circumstances provided for in the General Social Security Law (LGSS).

**Supplementary Table 1. Occupational exposure limit values.**

(1) CE Number, i.e., EINECS, ELINCS, or "no-longer polymer" (NLP), is the official number of the substance in the European Union, as defined in section 1.1.1.2 of Annex VI, Part 1, of Regulation (EC) No. 1272/2008. (2) CAS Number: Chemical Abstracts Service registry number. (3) Measured or calculated as a time-weighted average over an eight-hour reference period. (4) Short-term exposure limit: the limit value above which exposure should not occur, referring to a 15-minute period unless otherwise specified. (5) mg/m³ = milligrams per cubic meter of air at 20 °C and 101.3 kPa (760 mm Hg). (6) ppm = parts per million by volume of air (ml/m³). (7) f/ml = fibers per milliliter. (8) Inhalable fraction: if hardwood dust is mixed with dust from other woods, the limit value applies to the total wood dust present in the mixture. (9) Respirable fraction. (10) Potential significant contribution to total body burden via dermal exposure.

| **Agent Name** | **CE No. (1)** | **CAS No. (2)** | **Limit values** | | | | | | **Remarks** | **Transitional Measures** |
| --- | --- | --- | --- | --- | --- | --- | --- | --- | --- | --- |
|  |  |  | **Daily exposure (3)** | | | **Short-term exposure (4)** | | |  |  |
|  |  |  | **mg/m3 (5)** | **ppm (6)** | **f/ml (7)** | **mg/m3 (5)** | **ppm (6)** | **f/ml (7)** |  |  |
| **Hard wood dust** | – | – | 2 (8) | – | -– | – | – | – | – | Limit value: 3 mg/m³ until 17 January 2023 |
| **Chromium VI compounds classified as carcinogens under Article 2.1 of this Royal Decree (expressed as chromium)** | – | – | 0,005 | – | – | – | – | – | – | \|  \| \| --- \|   Limit value: 0.010 mg/m³ until 17 January 2025. Limit value: 0.025 mg/m³ for welding, plasma cutting or similar processes generating fumes, until 17 January 2025 |
| **Refractory ceramic fibers classified as carcinogens under Article 2.1 of this Royal Decree** | – | – | – | – | 0,3 | – | – | – | – |  |
| **Respirable crystalline silica dust** | – | – | 0,05 (9) | – | – | – | – | – | – | Limit value: 0.1 mg/m³ until 31 December 2021 |
| **Benzene** | 200-753-7 | 71-43-2 | 3,25 | 1 | – | – | – | – | Skin (10) |  |
| **Vinyl chloride monomer** | 200-831-0 | 75-01-4 | 2,6 | 1 | – | – | – | – | – |  |
| **Ethylene oxide** | 200-849-9 | 75-21-8 | 1,8 | 1 | – | – | – | – | Skin (10) |  |
| **1,2-Epoxypropane** | 200-879-2 | 75-56-9 | 2,4 | 1 | – | – | – | – | – |  |
| **Trichloroethylene** | 201-167-4 | 79-01-6 | 54,7 | 10 | - | 164,1 | 30 | - | Skin (10) |  |
| **Acrylamide** | 201-173-7 | 79-06-1 | 0,03 | – | – | – | – | – | Skin (10) |  |
| **2-Nitropropane** | 201-209-1 | 79-46-9 | 18 | 5 | – | – | – | – | – |  |
| **o-Toluidine** | 202-429-0 | 95-53-4 | 0,5 | 0,1 | – | – | – | – | Skin (10) |  |
| **4,4′-Methylenedianiline** | 202-974-4 | 101-77-9 | 0,08 | – | – | – | – | – | Skin (10) |  |
| **Epichlorohydrin** | 203-439-8 | 106-89-8 | 1,9 | – | – | – | – | – | Skin (10) |  |
| **Ethylene dibromide** | 203-444-5 | 106-93-4 | 0,8 | 0,1 | – | – | – | – | Skin (10) |  |
| **1,3-Butadiene** | 203-450-8 | 106-99-0 | 2,2 | 1 | – | – | – | – | – |  |
| **Ethylene dichloride** | 203-458-1 | 107-06-2 | 8,2 | 2 | – | – | – | – | Skin (10) |  |
| **Hydrazine** | 206-114-9 | 302-01-2 | 0,013 | 0,01 | – | – | – | – | Skin (10) |  |
| **Bromoethylene** | 209-800-6 | 593-60-2 | 2,2 | 0,5 | – | – | – | – | – |  |
| **Diesel engine exhaust emissions** |  |  | 0,05 (*) (9) |  |  |  |  |  |  | Limit applies from 21 February 2023. For underground mining and tunnel construction, from 21 February 2026 |
| **Polycyclic aromatic hydrocarbons mixtures, particularly those containing benzo[a]pyrene and classified as carcinogens under this Royal Decree** |  |  |  |  |  |  |  |  | Skin (10) |  |
| **Used mineral oils from internal combustion engines used for lubricating and cooling engine moving parts** |  |  |  |  |  |  |  |  | Skin (10) |  |
| **Cadmium and its inorganic compounds** |  |  | 0,001 |  |  |  |  |  |  | Limit value: 0.002 mg/m³ (11) until 11 July 2027 |
| **Beryllium and its inorganic compounds** |  |  | 0,0002 |  |  |  |  |  | Skin and respiratory sensitization (12) |  |
| **Arsenic acid and its salts, and inorganic arsenic compounds** |  |  | 0,01 |  |  |  |  |  |  |  |
| **Formaldehyde** | 200-001-8 | 50-00-0 | 0,37 | 0,3 |  | 0,74 | 0,6 |  | Skin sensitization (13) |  |
| **4,4′-Methylenebis(2-chloroaniline)** | 202-918-9 | 101-14-4 | 0,01 |  |  |  |  |  | Skin (10) |  |
| **Acrylonitrile** | 203-466-5 | 107-13-1 | 1 | 0,45 |  | 4 | 1,8 |  | Skin, dermal sensitization (13) | Limit value: 2 ppm (4.4 mg/m³) until 5 April 2026 |
| **Nickel compounds (as nickel)** |  |  | 0,01 (9) |  |  |  |  |  | Dermal and respiratory sensitization (12) | Limit value (9) applies from 18 January 2025 |
|  |  |  | 0,05 |  |  |  |  |  |  | Limit applies from 18 January 2025. Until then: 0.1 mg/m³ |
| **Lead and its inorganic compounds** |  |  | 0,15 |  |  |  |  |  |  |  |
| **N,N-Dimethylformamide** | 204-826-4 | 127-19-5 | 36 | 10 |  | 72 | 20 |  | Piel (10) |  |
| **Nitrobenzene** | 202-716-0 | 98-95-3 | 1 | 0,2 |  |  |  |  | Piel (10) |  |
| **N,N-Dimetilformamida.** | 200-679-5 | 68-12-2 | 15 | 5 |  | 30 | 10 |  | Piel (10) |  |
| **2-Methoxyethanol** | 203-713-7 | 109-86-4 |  | 1 |  |  |  |  | Piel (10) |  |
| **2-Methoxyethyl acetate** | 203-772-9 | 110-49-6 |  | 1 |  |  |  |  | Piel (10) |  |
| **2-Ethoxyethanol** | 203-804-1 | 110-80-5 | 8 | 2 |  |  |  |  | Piel (10) |  |
| **2-Ethoxyethyl acetate** | 203-839-2 | 111-15-9 | 11 | 2 |  |  |  |  | Piel (10) |  |
| **1-Methyl-2-pyrrolidone** | 212-828-1 | 872-50-4 | 40 | 10 |  | 80 | 20 |  | Piel (10) |  |
| **Mercury and inorganic divalent mercury compounds (including mercury oxide and mercury chloride, as mercury)** |  |  | 0,02 |  |  |  |  |  |  |  |
| **Bisphenol A; 4,4’-Isopropylidenediphenol** | 201-245-8 | 80-05-7 | 2 |  |  |  |  |  |  |  |
| **Carbon monoxide** | 211-128-3 | 630-08-0 | 23 | 20 |  | 117 | 100 |  |  |  |

**Supplementary Table 2. Process for Reporting Suspected Occupational Disease by Autonomous Community (AC).**
Excel file.

**Supplementary Figure 1. Responsibilities in Temporary Disability Processes.
Source:** General Social Security Law (LGSS). *INSS: National Institute of Social Security.

**Legend:** Temporary Disability (TD); National Institute of Social Security (INSS); Public Health Service (SPS).

**Supplementary Figure 2.** Basic concepts and degrees of permanent disability.
Source: Royal Decree 8/2015 of October 30, which approves the revised text of the General Social Security Law (Official State Gazette No. 261, October 31, 2015. Reference: BOE-A-2015-11724).
a Permanent disability generally arises from a prior period of temporary disability, except in very specific circumstances provided for in the General Social Security Law (LGSS).
